# *N*-(3-Bromo-5-methyl-2-pyrid­yl)-4-methyl­benzene­sulfonamide

**DOI:** 10.1107/S1600536809054014

**Published:** 2009-12-19

**Authors:** Ming Peng, Youfu Luo, Lijuan Chen

**Affiliations:** a State Key Laboratory of Biotherapy, Sichuan University, Chengdu 610064, People’s Republic of China

## Abstract

In the mol­ecule of the title compound, C_13_H_13_BrN_2_O_2_S, the dihedral angle formed by the pyridine and benzene rings is 66.87 (3)°. An intra­molecular N—H⋯Br hydrogen bond is observed. In the crystal structure, N—H⋯O hydrogen bonds, C—H⋯π inter­actions and aromatic π–π stacking inter­actions [centroid–centroid distance = 3.757 (14) Å] link the mol­ecules into a three-dimensional network.

## Related literature

The title compound is a key inter­mediate in the synthesis of new anti­tumor drugs including TGX221 [systematic name 7-methyl-2-(4-morpholinyl)-9-[1-(phenylamino)ethyl]-4*H*-pyrido[1,2-*a*]pyrimidin-4-one]. For the biological activity of TGX221, see: Jackson *et al.* (2005 ▶).
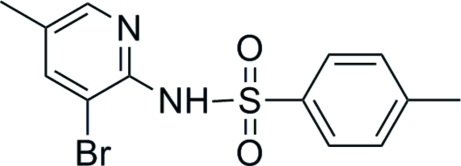


## Experimental

### 

#### Crystal data

C_13_H_13_BrN_2_O_2_S
*M* *_r_* = 341.22
Monoclinic, 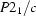

*a* = 11.832 (2) Å
*b* = 13.305 (3) Å
*c* = 8.6263 (17) Å
β = 105.52 (3)°
*V* = 1308.5 (5) Å^3^
*Z* = 4
Mo *K*α radiation
μ = 3.30 mm^−1^
*T* = 113 K
0.22 × 0.21 × 0.18 mm

#### Data collection

Rigaku Saturn CCD area-detector diffractometer
Absorption correction: multi-scan (*CrystalClear*; Rigaku/MSC, 2005 ▶) *T*_min_ = 0.531, *T*_max_ = 0.588
10637 measured reflections
3102 independent reflections
2455 reflections with *I* > 2σ(*I*)
*R*_int_ = 0.037

#### Refinement

*R*[*F* ^2^ > 2σ(*F* ^2^)] = 0.028
*wR*(*F* ^2^) = 0.071
*S* = 1.03
3102 reflections
178 parameters
H atoms treated by a mixture of independent and constrained refinement
Δρ_max_ = 0.38 e Å^−3^
Δρ_min_ = −0.66 e Å^−3^

### 

Data collection: *DIFRAC* (Gabe & White, 1993 ▶); cell refinement: *DIFRAC*; data reduction: *NRCVAX* (Gabe *et al.*, 1989 ▶); program(s) used to solve structure: *SHELXS97* (Sheldrick, 2008 ▶); program(s) used to refine structure: *SHELXL97* (Sheldrick, 2008 ▶); molecular graphics: *ORTEPIII* (Burnett & Johnson, 1996 ▶) and *Mercury* (Macrae *et al.*, 2006 ▶); software used to prepare material for publication: *SHELXL97*.

## Supplementary Material

Crystal structure: contains datablocks global, I. DOI: 10.1107/S1600536809054014/rz2394sup1.cif

Structure factors: contains datablocks I. DOI: 10.1107/S1600536809054014/rz2394Isup2.hkl

Additional supplementary materials:  crystallographic information; 3D view; checkCIF report

## Figures and Tables

**Table 1:** Hydrogen-bond geometry (Å, °) *Cg*2 is is the centroid of the N2, C1–C5 ring.

*D*—H⋯*A*	*D*—H	H⋯*A*	*D*⋯*A*	*D*—H⋯*A*
N1—H1⋯Br1	0.72 (3)	2.78 (2)	3.134 (2)	114 (2)
N1—H1⋯O1^i^	0.72 (3)	2.53 (3)	3.225 (2)	164 (3)
C3—H3⋯*Cg*1^ii^	0.95	2.76	3.648 (2)	155

Symmetry codes: (i) 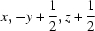; (ii) 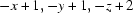.

**Fig. 1.**
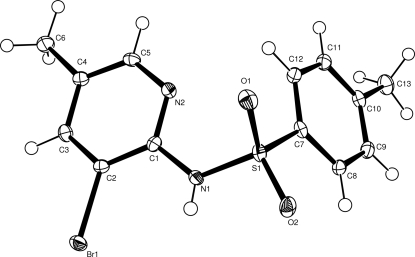
The molecular structure of title compound showing 30% probability displacement ellipsoids and the atom-numbering scheme.

## supplementary crystallographic information

### Comment

TGX221, a selective inhibitor of PI3K p110β, and its derivatives are of great importance owing to their wide biological properties (Jackson *et al.*, 2005). We report herein the crystal structure of the title compound, which is one of the key intermediates in our synthetic investigations of new antitumor drugs.

The molecular structure of the title compound is shown in Fig. 1. The dihedral angle between the benzene and pyridine is 66.87 (3)°. The molecular conformation is stabilized by an intramolecular N—H···Br hydrogen bond (Table 1). In the crystal, molecules are linked into a three-dimensional network by intermolecular N—H···O hydrogen bonds and aromatic π–π stacking interactions involving centrosymmetrically related pyridine and benzene rings, with a centroid-to-centroid distance of 3.757 (14) Å. In addition, C—H···π interactions are also present (Table 1; Cg1 is the centroid of the C7–C12 benzene ring).

### Experimental

A mixture of 3-bromo-5-methylpyridin-2-amine (18.6 g, 0.1 mol), 4-methylbenzene-1-sulfonyl chloride (38.2 g, 0.2 mol), and pyridine (7.9 g, 0.10 mol), as a catalyst were charged into a three-necked round-bottomed flask fitted with a mechanical stirrer, a thermometer and a nitrogen inlet. The mixture was stirred vigorously at 100°C for 3 h. After the reactor was cooled to room temperature, the reaction solution was poured into water. The resulting solid was filtered, washed with water, dried and recrystallized from the mixture of hexane:ethyl acetate (3:1 v(v) to get colourless crystals suitable for X-ray analysis.

### Refinement

The amine H atom was located in a difference Fourier map and refined freely. All other H atoms were positioned geometrically and refined using a riding model, with C—H = 0.95–0.98 Å and with *U*_iso_(H) = 1.2 *U*_eq_(C) or 1.5*U*_eq_(C) for methyl H atoms.

### Crystal data

**Table:**

C_13_H_13_BrN_2_O_2_S	*F*(000) = 688
*M**_r_* = 341.22	*D*_x_ = 1.732 Mg m^−^^3^
Monoclinic, *P*2_1_/*c*	Mo *K*α radiation, λ = 0.71073 Å
Hall symbol: -P 2ybc	Cell parameters from 4245 reflections
*a* = 11.832 (2) Å	θ = 2.3–27.9°
*b* = 13.305 (3) Å	µ = 3.30 mm^−^^1^
*c* = 8.6263 (17) Å	*T* = 113 K
β = 105.52 (3)°	Block, colourless
*V* = 1308.5 (5) Å^3^	0.22 × 0.21 × 0.18 mm
*Z* = 4	

### Data collection

**Table:**

Rigaku Saturn CCD area-detector diffractometer	3102 independent reflections
Radiation source: rotating anode	2455 reflections with *I* > 2σ(*I*)
confocal	*R*_int_ = 0.037
Detector resolution: 7.31 pixels mm^-1^	θ_max_ = 27.9°, θ_min_ = 2.4°
φ and ω scans	*h* = −9→15
Absorption correction: multi-scan (*CrystalClear*; Rigaku/MSC, 2005)	*k* = −17→17
*T*_min_ = 0.531, *T*_max_ = 0.588	*l* = −10→11
10637 measured reflections	

### Refinement

**Table:**

Refinement on *F*^2^	Primary atom site location: structure-invariant direct methods
Least-squares matrix: full	Secondary atom site location: difference Fourier map
*R*[*F*^2^ > 2σ(*F*^2^)] = 0.028	Hydrogen site location: mixed
*wR*(*F*^2^) = 0.071	H atoms treated by a mixture of independent and constrained refinement
*S* = 1.03	*w* = 1/[σ^2^(*F*_o_^2^) + (0.0398*P*)^2^] where *P* = (*F*_o_^2^ + 2*F*_c_^2^)/3
3102 reflections	(Δ/σ)_max_ = 0.001
178 parameters	Δρ_max_ = 0.38 e Å^−^^3^
0 restraints	Δρ_min_ = −0.66 e Å^−^^3^

### Special details

**Table:**

Geometry. All e.s.d.'s (except the e.s.d. in the dihedral angle between two l.s. planes) are estimated using the full covariance matrix. The cell e.s.d.'s are taken into account individually in the estimation of e.s.d.'s in distances, angles and torsion angles; correlations between e.s.d.'s in cell parameters are only used when they are defined by crystal symmetry. An approximate (isotropic) treatment of cell e.s.d.'s is used for estimating e.s.d.'s involving l.s. planes.
Refinement. Refinement of *F*^2^ against ALL reflections. The weighted *R*-factor *wR* and goodness of fit *S* are based on *F*^2^, conventional *R*-factors *R* are based on *F*, with *F* set to zero for negative *F*^2^. The threshold expression of *F*^2^ > σ(*F*^2^) is used only for calculating *R*-factors(gt) *etc*. and is not relevant to the choice of reflections for refinement. *R*-factors based on *F*^2^ are statistically about twice as large as those based on *F*, and *R*- factors based on ALL data will be even larger.

### Fractional atomic coordinates and isotropic or equivalent isotropic displacement parameters (Å^2^)

**Table:**

	*x*	*y*	*z*	*U*_iso_*/*U*_eq_	
Br1	0.542001 (19)	0.267131 (17)	1.09928 (3)	0.01935 (8)	
S1	0.23555 (5)	0.35572 (4)	0.64144 (6)	0.01313 (13)	
O1	0.27402 (13)	0.33591 (11)	0.50006 (17)	0.0180 (3)	
O2	0.14905 (13)	0.29181 (11)	0.67992 (18)	0.0174 (3)	
N1	0.34782 (16)	0.34723 (14)	0.8024 (2)	0.0142 (4)	
H1	0.339 (2)	0.312 (2)	0.861 (3)	0.026 (8)*	
N2	0.46548 (15)	0.46217 (13)	0.7147 (2)	0.0143 (4)	
C1	0.45737 (17)	0.39383 (15)	0.8237 (2)	0.0118 (4)	
C2	0.55266 (18)	0.36978 (14)	0.9520 (2)	0.0119 (4)	
C3	0.65784 (18)	0.42026 (15)	0.9708 (2)	0.0146 (4)	
H3	0.7229	0.4056	1.0598	0.018*	
C4	0.66762 (18)	0.49303 (15)	0.8579 (2)	0.0137 (4)	
C5	0.56853 (19)	0.50966 (15)	0.7326 (2)	0.0146 (4)	
H5	0.5736	0.5580	0.6537	0.018*	
C6	0.77966 (19)	0.55118 (16)	0.8732 (3)	0.0194 (5)	
H6A	0.7756	0.5862	0.7719	0.029*	
H6B	0.8463	0.5047	0.8971	0.029*	
H6C	0.7898	0.6004	0.9604	0.029*	
C7	0.18506 (18)	0.48129 (15)	0.6312 (2)	0.0131 (4)	
C8	0.09300 (18)	0.50427 (16)	0.6980 (2)	0.0135 (4)	
H8	0.0581	0.4534	0.7471	0.016*	
C9	0.05321 (18)	0.60313 (16)	0.6914 (2)	0.0158 (4)	
H9	−0.0099	0.6193	0.7356	0.019*	
C10	0.10400 (19)	0.67832 (15)	0.6215 (2)	0.0150 (4)	
C11	0.19340 (19)	0.65226 (16)	0.5508 (3)	0.0166 (5)	
H11	0.2269	0.7027	0.4990	0.020*	
C12	0.23404 (18)	0.55464 (16)	0.5549 (2)	0.0154 (4)	
H12	0.2947	0.5380	0.5061	0.018*	
C13	0.0635 (2)	0.78579 (17)	0.6201 (3)	0.0216 (5)	
H13A	0.1296	0.8283	0.6755	0.032*	
H13B	0.0012	0.7903	0.6753	0.032*	
H13C	0.0332	0.8086	0.5087	0.032*	

### Atomic displacement parameters (Å^2^)

**Table:**

	*U*^11^	*U*^22^	*U*^33^	*U*^12^	*U*^13^	*U*^23^
Br1	0.01556 (13)	0.02025 (13)	0.02032 (14)	−0.00270 (9)	0.00147 (9)	0.00962 (9)
S1	0.0115 (3)	0.0124 (2)	0.0134 (3)	0.0010 (2)	−0.0002 (2)	−0.00113 (19)
O1	0.0207 (8)	0.0192 (8)	0.0133 (8)	0.0031 (7)	0.0029 (7)	−0.0030 (6)
O2	0.0123 (8)	0.0144 (7)	0.0235 (9)	−0.0028 (6)	0.0011 (7)	0.0003 (6)
N1	0.0114 (9)	0.0164 (9)	0.0135 (10)	−0.0018 (8)	0.0009 (8)	0.0047 (8)
N2	0.0138 (9)	0.0157 (9)	0.0130 (8)	0.0020 (7)	0.0026 (7)	0.0018 (7)
C1	0.0119 (10)	0.0119 (10)	0.0124 (10)	0.0006 (8)	0.0047 (8)	−0.0027 (8)
C2	0.0144 (10)	0.0104 (9)	0.0119 (10)	−0.0005 (8)	0.0051 (8)	−0.0003 (8)
C3	0.0130 (10)	0.0157 (10)	0.0128 (11)	0.0001 (9)	−0.0005 (9)	−0.0014 (8)
C4	0.0156 (11)	0.0120 (10)	0.0139 (10)	−0.0011 (9)	0.0049 (9)	−0.0005 (8)
C5	0.0187 (11)	0.0115 (10)	0.0148 (10)	−0.0006 (9)	0.0063 (9)	0.0027 (8)
C6	0.0170 (12)	0.0192 (11)	0.0218 (12)	−0.0034 (10)	0.0051 (9)	0.0039 (9)
C7	0.0121 (10)	0.0133 (10)	0.0115 (10)	0.0018 (8)	−0.0008 (8)	0.0001 (8)
C8	0.0108 (10)	0.0167 (10)	0.0123 (10)	−0.0011 (9)	0.0019 (8)	0.0001 (8)
C9	0.0117 (10)	0.0215 (11)	0.0140 (11)	0.0019 (9)	0.0031 (9)	−0.0023 (9)
C10	0.0141 (11)	0.0141 (11)	0.0138 (11)	0.0017 (9)	−0.0015 (9)	−0.0011 (8)
C11	0.0138 (11)	0.0173 (11)	0.0177 (11)	−0.0007 (9)	0.0026 (9)	0.0025 (9)
C12	0.0120 (10)	0.0201 (11)	0.0141 (11)	0.0013 (9)	0.0036 (9)	0.0008 (8)
C13	0.0206 (12)	0.0172 (12)	0.0242 (13)	0.0015 (9)	0.0009 (10)	−0.0009 (9)

### Geometric parameters (Å, °)

**Table:**

Br1—C2	1.8925 (19)	C6—H6B	0.9800
S1—O1	1.4357 (15)	C6—H6C	0.9800
S1—O2	1.4361 (15)	C7—C12	1.388 (3)
S1—N1	1.649 (2)	C7—C8	1.396 (3)
S1—C7	1.768 (2)	C8—C9	1.393 (3)
N1—C1	1.404 (3)	C8—H8	0.9500
N1—H1	0.72 (2)	C9—C10	1.385 (3)
N2—C1	1.330 (2)	C9—H9	0.9500
N2—C5	1.345 (3)	C10—C11	1.399 (3)
C1—C2	1.389 (3)	C10—C13	1.507 (3)
C2—C3	1.385 (3)	C11—C12	1.382 (3)
C3—C4	1.400 (3)	C11—H11	0.9500
C3—H3	0.9500	C12—H12	0.9500
C4—C5	1.384 (3)	C13—H13A	0.9800
C4—C6	1.510 (3)	C13—H13B	0.9800
C5—H5	0.9500	C13—H13C	0.9800
C6—H6A	0.9800		
			
O1—S1—O2	119.68 (9)	C4—C6—H6C	109.5
O1—S1—N1	109.63 (9)	H6A—C6—H6C	109.5
O2—S1—N1	103.19 (9)	H6B—C6—H6C	109.5
O1—S1—C7	108.09 (9)	C12—C7—C8	120.86 (19)
O2—S1—C7	108.59 (10)	C12—C7—S1	120.64 (16)
N1—S1—C7	106.98 (9)	C8—C7—S1	118.48 (15)
C1—N1—S1	125.99 (15)	C9—C8—C7	118.85 (19)
C1—N1—H1	120 (2)	C9—C8—H8	120.6
S1—N1—H1	113 (2)	C7—C8—H8	120.6
C1—N2—C5	118.33 (18)	C10—C9—C8	121.23 (19)
N2—C1—C2	121.59 (19)	C10—C9—H9	119.4
N2—C1—N1	116.62 (18)	C8—C9—H9	119.4
C2—C1—N1	121.79 (18)	C9—C10—C11	118.52 (19)
C3—C2—C1	119.62 (18)	C9—C10—C13	120.96 (19)
C3—C2—Br1	119.36 (15)	C11—C10—C13	120.53 (19)
C1—C2—Br1	121.01 (15)	C12—C11—C10	121.4 (2)
C2—C3—C4	119.51 (19)	C12—C11—H11	119.3
C2—C3—H3	120.2	C10—C11—H11	119.3
C4—C3—H3	120.2	C11—C12—C7	119.1 (2)
C5—C4—C3	116.35 (19)	C11—C12—H12	120.5
C5—C4—C6	121.78 (18)	C7—C12—H12	120.5
C3—C4—C6	121.87 (19)	C10—C13—H13A	109.5
N2—C5—C4	124.57 (19)	C10—C13—H13B	109.5
N2—C5—H5	117.7	H13A—C13—H13B	109.5
C4—C5—H5	117.7	C10—C13—H13C	109.5
C4—C6—H6A	109.5	H13A—C13—H13C	109.5
C4—C6—H6B	109.5	H13B—C13—H13C	109.5
H6A—C6—H6B	109.5		
			
O1—S1—N1—C1	49.3 (2)	C6—C4—C5—N2	−178.28 (18)
O2—S1—N1—C1	177.92 (17)	O1—S1—C7—C12	−31.16 (19)
C7—S1—N1—C1	−67.63 (19)	O2—S1—C7—C12	−162.43 (16)
C5—N2—C1—C2	−1.2 (3)	N1—S1—C7—C12	86.82 (18)
C5—N2—C1—N1	178.89 (17)	O1—S1—C7—C8	147.25 (15)
S1—N1—C1—N2	10.4 (3)	O2—S1—C7—C8	15.98 (18)
S1—N1—C1—C2	−169.47 (15)	N1—S1—C7—C8	−94.77 (17)
N2—C1—C2—C3	2.3 (3)	C12—C7—C8—C9	−2.0 (3)
N1—C1—C2—C3	−177.83 (18)	S1—C7—C8—C9	179.57 (15)
N2—C1—C2—Br1	−176.47 (15)	C7—C8—C9—C10	−0.6 (3)
N1—C1—C2—Br1	3.4 (3)	C8—C9—C10—C11	2.8 (3)
C1—C2—C3—C4	−1.7 (3)	C8—C9—C10—C13	−177.77 (19)
Br1—C2—C3—C4	177.10 (14)	C9—C10—C11—C12	−2.4 (3)
C2—C3—C4—C5	0.1 (3)	C13—C10—C11—C12	178.16 (19)
C2—C3—C4—C6	179.38 (19)	C10—C11—C12—C7	−0.2 (3)
C1—N2—C5—C4	−0.4 (3)	C8—C7—C12—C11	2.4 (3)
C3—C4—C5—N2	1.0 (3)	S1—C7—C12—C11	−179.22 (16)

### Hydrogen-bond geometry (Å, °)

**Table:**

Cg2 is is the centroid of the N2, C1–C5 ring.

**Table:**

*D*—H···*A*	*D*—H	H···*A*	*D*···*A*	*D*—H···*A*
N1—H1···Br1	0.72 (3)	2.78 (2)	3.134 (2)	114 (2)
N1—H1···O1^i^	0.72 (3)	2.53 (3)	3.225 (2)	164 (3)
C3—H3···Cg1^ii^	0.95	2.76	3.648 (2)	155

Symmetry codes: (i) *x*, −*y*+1/2, *z*+1/2; (ii) −*x*+1, −*y*+1, −*z*+2.
